# The ChIP-seq-Defined Networks of Bcl-3 Gene Binding Support Its Required Role in Skeletal Muscle Atrophy

**DOI:** 10.1371/journal.pone.0051478

**Published:** 2012-12-10

**Authors:** Robert W. Jackman, Chia-Ling Wu, Susan C. Kandarian

**Affiliations:** Department of Health Sciences, Boston University, Boston, Massachusetts, United States of America; Università di Milano, Italy

## Abstract

NF-kappaB transcriptional activation is required for skeletal muscle disuse atrophy. We are continuing to study how the activation of NF-kB regulates the genes that encode the protein products that cause atrophy. Using ChIP-sequencing we found that Bcl-3, an NF-kB transcriptional activator required for atrophy, binds to the promoters of a number of genes whose collective function describes two major aspects of muscle wasting. By means of bioinformatics analysis of ChIP-sequencing data we found Bcl-3 to be directing transcription networks of proteolysis and energy metabolism. The proteolytic arm of the Bcl-3 networks includes many E3 ligases associated with proteasomal protein degradation, including that of the N-end rule pathway. The metabolic arm appears to be involved in organizing the change from oxidative phosphorylation to glycolysis in atrophying muscle. For one gene, MuRF1, ChIP-sequencing data identified the location of Bcl-3 and p50 binding in the promoter region which directed the creation of deletant and base-substitution mutations of MuRF1 promoter constructs to determine the effect on gene transcription. The results provide the first direct confirmation that the NF-kB binding site is involved in the muscle unloading regulation of MuRF1. Finally, we have combined the ChIP-sequencing results with gene expression microarray data from unloaded muscle to map several direct targets of Bcl-3 that are transcription factors whose own targets describe a set of indirect targets for NF-kB in atrophy. ChIP-sequencing provides the first molecular explanation for the finding that Bcl3 knockout mice are resistant to disuse muscle atrophy. Mapping the transcriptional regulation of muscle atrophy requires an unbiased analysis of the whole genome, which we show is now possible with ChIP-sequencing.

## Introduction

Skeletal muscle atrophy is the result of a metabolic shift that increases the rate of proteolysis and/or decreases the rate protein synthesis in the cells that make up muscle. The initiating triggers for this shift are varied, but fall into two main categories: the result of a disease or pathology such as cancer, diabetes, HIV, major body burns, and sepsis, or the loss of muscle as a result of immobilization, bed rest, diaphragm breathing assistance, or decreases in gravity as in space travel [1], [2], [3], [4]. Since the triggers of atrophy differ it might be expected that there are differences in the cellular processes that control disuse and disease-induced muscle atrophy [5], [6].

Investigations into the signaling pathways activated by muscle disuse due to the removal of weight bearing (i.e., unloading) discovered that nuclear factor-kappaB (NF-kB) activity was increased early and continuously [7], [8], [9]. The NF-kB transcription factors showing increased localization to the muscle cell nuclei were p50 and Bcl-3, but not p65 [7], [10]. Viable knockouts of genes for these two proteins made possible the finding that the elimination of either gene alone would block muscle atrophy due to unloading [8]. To identify the genes regulated by p50 or Bcl-3 that produce the atrophied phenotype, global gene expression analysis was used to compare wild type and the two knockout strains of mice in response to unloading [10]. The genes upregulated in wild type mice that were not upregulated in knockout mice due to unloading were from several muscle atrophy gene functional groups including proteolysis. However this analysis cannot distinguish direct vs. indirect target genes.

**Figure 1:**
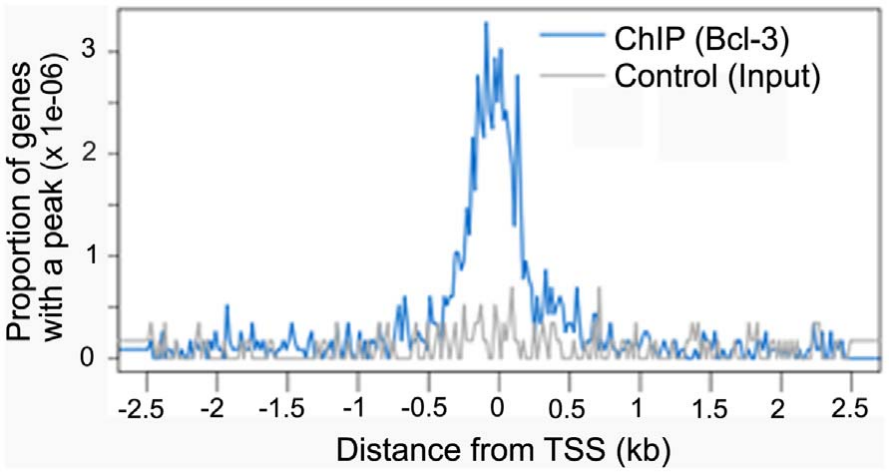
Distribution of Bcl-3 binding peaks around transcription start sites (TSS) determined by Nebula/Galaxy. Blue represents the plot of Bcl-3 peaks from unloaded muscle and gray represents the plot from peaks found in the input chromatin from unloaded muscle. The y-axis is the proportion of peaks relative to all genes in the genome. Peaks are plotted every 20 bases from −2500 to +2500 relative to the TSS.

**Figure 2:**
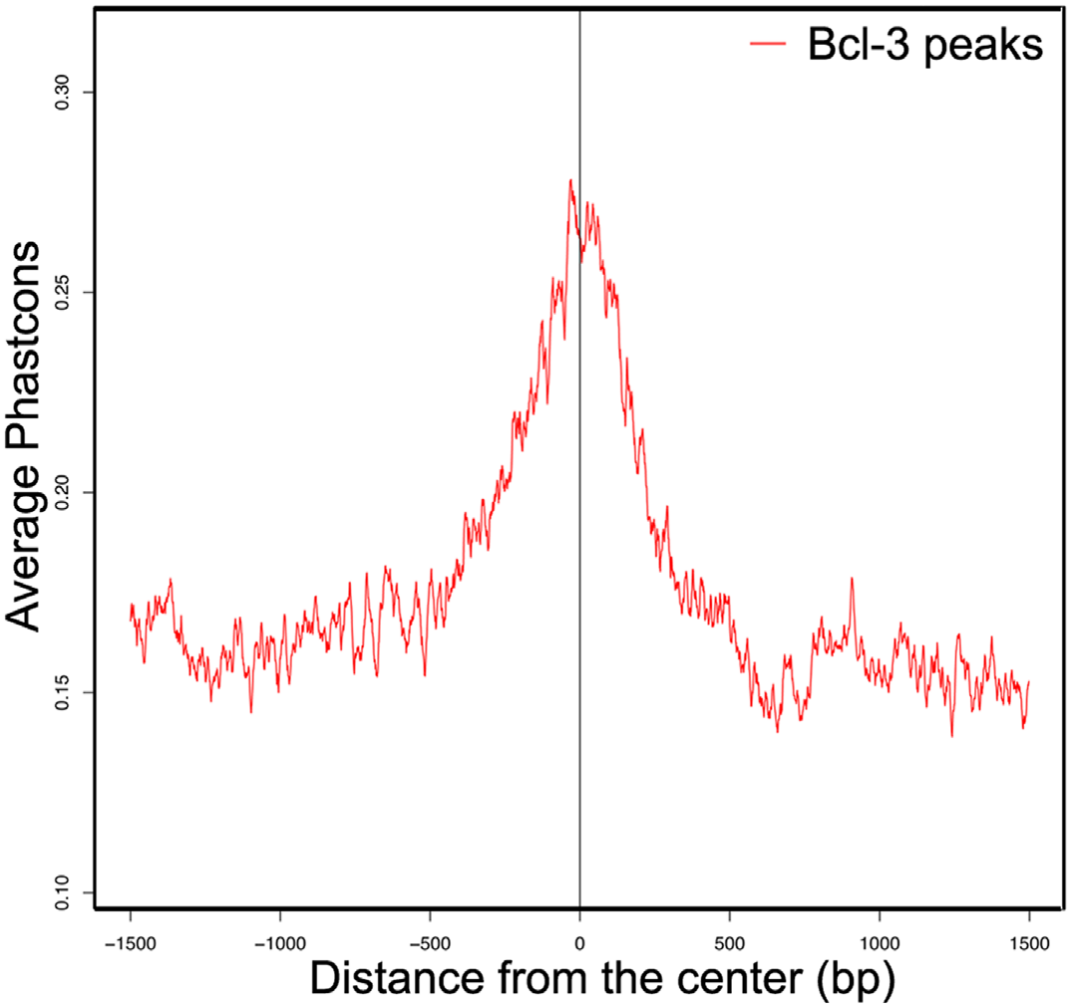
Plot of phylogenomic conservation for the 2,817 Bcl-3 peaks produced by unloading. The peaks and surrounding genome regions (−1500 bp to +1500 bp) were compared to a database of Phastcon alignment scores for 31 placental mammals on the Galaxy/Cistrome server. Phastcon scores are higher for sequence similarity and are weighted higher for species farther removed from mice phylogenetically. A Phastcon score of 1.0 would reflect perfect identity in all 31 species. Conservation is highest at the center of the peaks indicating that the centers share sequence homology between species, a sign that the sites of Bcl-3 binding are important to function.

In the present study, we focused on finding the direct target genes of NF-kB transcription factors during muscle unloading in order to identify the genes producing atrophy. We used chromatin immunoprecipitation followed by next generation sequencing (ChIP-seq), a recently developed method in which the location of particular transcription factors is mapped to the whole genome during physiological or pathological changes. The active molecules of NF-kB consist of a protein complex with two or more subunits. In the case of p50 and Bcl-3 the active molecule is thought to consist of a homodimer of p50, which contains the DNA recognition and binding activity, and a bound molecule of Bcl-3 which has two transactivation domains for the induction of gene expression [11], [12]. Using antibodies for p50 and Bcl-3 to immunoprecipitate the muscle chromatin followed by high-throughput sequencing and high-resolution genome mapping, we identified the genes that are being directly targeted by these NF-kB transcription factors in unloaded muscle. In addition, we identified the ontology pathways containing the genes found, providing evidence for the cellular functions organized by NF-kB in the process of muscle atrophy. Bioinformatic analysis showed that Bcl-3 is responsible for organizing the proteolytic genes that contribute to unloading atrophy. The pathways regulated by Bcl-3 also include those of the transition from aerobic to glycolytic metabolism in atrophying muscle. We have identified for the first time, gene target networks regulated by a transcription factor (Bcl-3) that is required for skeletal muscle atrophy.

**Figure 3:**
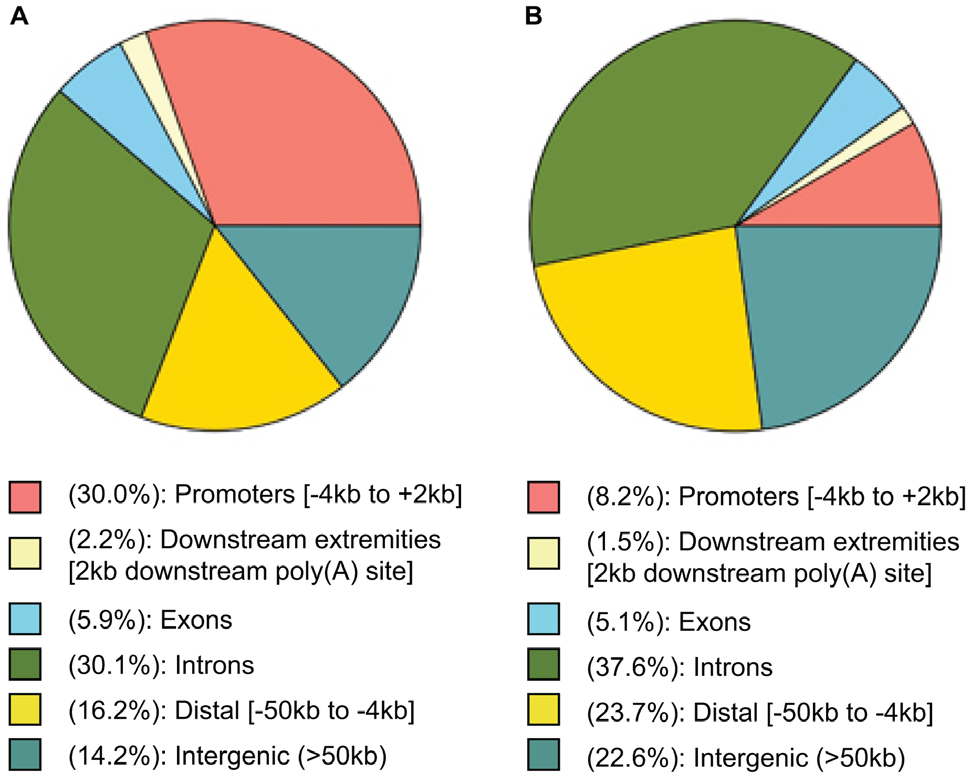
Distribution of Bcl-3 peaks by location in genes. (**A**) ChIPseeqer genomic annotation for the 2,817 peaks of increased Bcl-3 binding found in unloaded compared to control muscle. (**B**) ChIPseeqer genomic annotation for peaks found in the sequence alignments from the unloaded muscle input chromatin which was sheared and used to create a library without any further manipulation (no immunoprecipitation). The peak finder in ChIPseeqer was set to the same parameters as for the 2,817 Bcl-3 peaks in unloaded muscle and found 1,594 random peaks.

## Methods

### Animals and Hindlimb Unloading

For the gene expression array and for ChIP-seq, 7-week-old female wild type mice (C57BL/6J) were purchased from the Jackson Laboratory (Bar Harbor, ME). Animals were provided with chow and water ad libitum and housed individually in Boston University Animal Care Facility. After 3 days of acclimation, mice were randomly assigned to weight-bearing (WB) or hind limb unloaded (HU) groups. Mice in the HU group had their hind limbs elevated off the cage floor for 5 days to induce unloading induced muscle atrophy, as described previously [10]. We used published time course data from our microarray study [13] to identify an appropriate time point, when the most genes are differentially regulated, to use in undertaking a ChIP-seq study, and in this way to capture the time during the atrophy process that would best represent the time for binding of NF-kB transcription factors to the gene targets of the NF-kB transcriptional network.

For reporter activity measurements, 7-week-old female Wistar rats from Charles River Lab (Wilmington, MA) were used. 40 µg of wild type or mutant MuRF1-promoter reporters were transfected into rat soleus muscle as previously described [14]. Twenty four hours after reporter injection, rats were randomly assigned to either the weight bearing group or the HU group. The HU group of rats had their hind limbs removed from weight bearing for 5 days by elastic tail cast as described previously [14]. The use of animals in this study was approved by the Institutional Animal Care and Use Committee of Boston University (protocol number 12-012).

**Figure 4:**
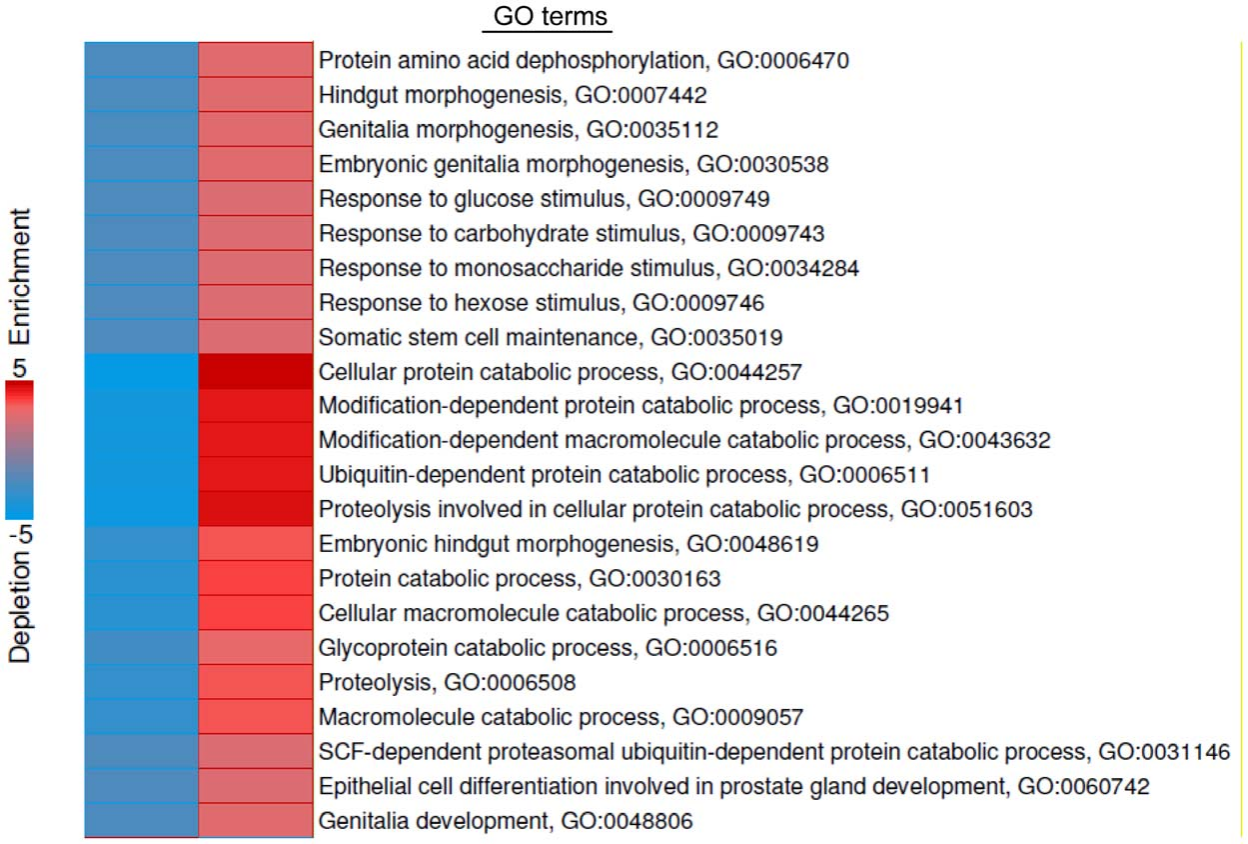
GO terms enriched in genes with Bcl-3 peaks during unloading. iPAGE analysis identified 23 GO terms over-represented (red bar) by genes with Bcl-3 peaks in promoters due to muscle unloading. Text labeling indicates the name of the GO term and the associated GO identification number.

### ChIP-seq

Gastrocnemius and plantaris muscles were isolated from weight bearing (i.e., control) or 5 day hind limb unloaded mice. Freshly dissected muscle was minced and cross-linked in 1% formaldehyde for 15 minutes, quenched with glycine and then frozen in liquid nitrogen. Tissues from four legs were pooled, homogenized, and chromatin isolated as we detailed previously [10]. This material was subjected to sonication to yield chromatin fragments that were on average 250 bp. An aliquot of sonicated chromatin was put aside to be used as the input fraction. The rest of the chromatin was diluted in IP buffer and split into groups for each antibody (Bcl-3 and p50) and one group without any primary antibody. The antibody treatments were for 16 hrs at 4°C with constant low speed mixing. The antibody-chromatin complexes were captured with Protein G magnetic beads. The chromatin was eluted from the beads and crosslinks reversed, followed by pronase/RNase treatment and precipitation of the DNA. One tenth of the material was used in PCR for genes already shown to give positive ChIP-PCR in order to test the ChIP. The different DNA libraries isolated from the ChIP with Bcl-3, p50, no antibody, and non-ChIP input chromatin were labeled for high throughput sequencing using the Illumina ChIP-seq Library kit. An aliquot of each library was examined by acrylamide electrophoresis and Sybr-gold staining to estimate the quality by size and intensity of the product which appears as a smear with average size of 250 bp. The libraries were sent to The Whitehead Institute (Cambridge, MA) where they were cleaned of adapter dimers using Ampure XL beads. The cleaned libraries were tested by Bioanalyzer and qPCR quality control was performed in order to determine how much of each library to use. The libraries were sequenced using Illumina Solexa sequencing on a GA II sequencer. The resulting sequences from control and unloaded samples were aligned to the mouse genome (mm9 version) using ELAND. The sequences were sent to our lab in the ELAND format.

**Table 1:** The genes from iPAGE ontology analysis.

GO category	Gene Name	Function
Protein catabolism (11 GO terms)	Adam17	Activates some membrane receptors
	Arih2	E3 ligase
	Ate1	Arginyl transferase
	Cul2	Component of ECS ubiquitination
	Fbxo6	E3 ligase
	Hspa5	Hsp 70 family member
	Itch	E3 ligase
	Ppt1	Lysosomal degradation
	Psen1	Intramembrane protein cleavage
	Rlim	Ring finger protein
	Sod1	Reactive radical destroyer
	Trim63	Muscle E3 ligase (MuRF1)
	Ubr1	n-recognin for N rule proteolysis
	Usp22	Ubiquitin thioesterase
Development (7 GO terms)	Apc	Wnt antagonist
	Psap	Sphingolipid recognition in lysosomes
	Tcf7l2	Wnt signaling glucose metabolism
	Eya3	Essential for myogenin activity
Glucose metabolism (4 GO terms)	Pfkl	phosphofructokinase
	Pygm	Glycogen phosphorylase
Phosphatases (1 GO term)	Dusp3	Phosphatase, MAPK inhibitor
	Ppm1g	phosphatase
	Ppp1cb	Phosphatase catalytic subunit
	Ppp1r12a	Regulation of Ppp1c

For the Bcl-3 ChIP we pooled two separate ChIP-seq experiments by combining.bam forms of the alignments. There were 40 million sequence reads in the control samples, of which 20.4 million were unique, and there were 52 million sequence reads in the unloaded samples of which 25.9 million were unique. The aligned sequences converted to.sam format were uploaded to the peak finder program in ChIPseeqer [15]. These alignment files were used for all subsequent analyses.

**Figure 5:**
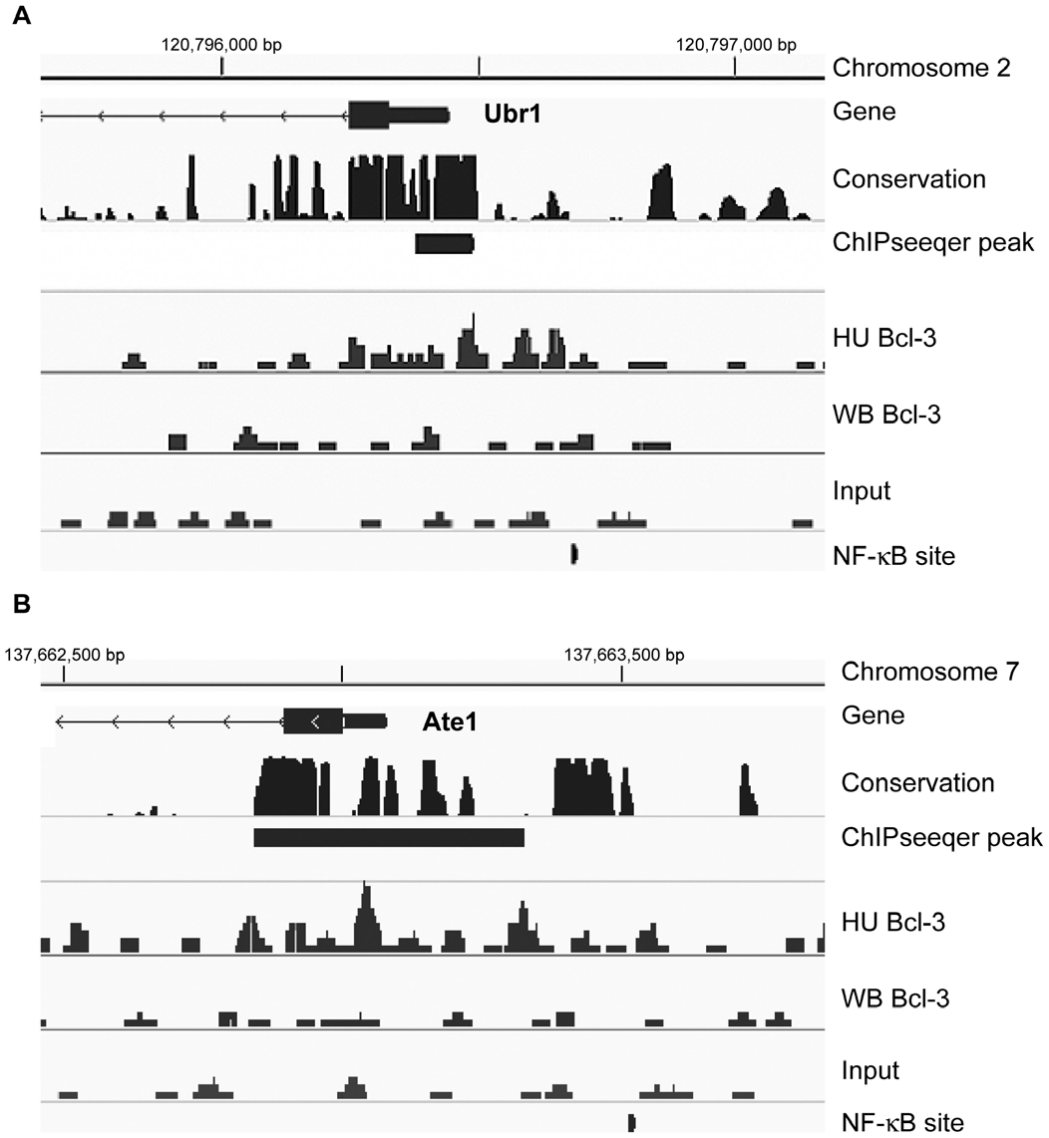
Bcl-3 binding profile at Ubr1 and Ate1 genes. (**A**) An assembly of ChIP-seq data for the Ubr1 (chromosome 2) and (**B**) Ate1 (chromosome 7) genes, visualized by IGV. In both **A** and **B**, the top line is a representation of genomic size and location of the region. Vertical ticks are 500 bp apart. The next rows are labeled as follows: Gene, the graphic for the name, location, and orientation for the gene nearest to the ChIP-seq alignment. The medium thick dark line is the 5′ utr of the gene and the thicker dark region is the first exon followed by a thin line with arrows which is intron 1; Conservation, the track of Phastcons for sequence similarity among placental mammals; ChIPseeqer peak, the black rectangular block is the location of the statistically-qualified peak of sequencing alignments called by the ChIPseeqer algorithm; HU Bcl-3, a representation of the.sam alignments for the Bcl-3 ChIPseq of the unloaded muscle; WB Bcl-3, a representation of the.sam alignments for the Bcl-3 ChIPseq of the weight bearing muscle; Input, a representation of the.sam alignments for non-ChIP unloaded chromatin; NF-κB site, location of a NF-κB consensus site identified by JASPAR.

**Table 2:** qPCR of selected proteolysis genes with increased Bcl-3 promoter binding.

Gene	Fold activation
Arih2	2.1
Ate1	1.5
Fbxo6	1.8
Itch	1.4
Rlim	1.6
Rnf13	1.5
Psmb7	1.9
Sod1	1.8
Trim63	2.0
Ubb	1.8
Ubr1	1.7

Fold change, control vs. unloaded.

### Total RNA Isolation and RT-qPCR

Gastrocnemius and plantaris muscles harvested from anesthetized wild type mice from control and HU groups (n = 6 per group) were snap frozen in liquid nitrogen and stored at −80°C before use. Total RNA was isolated using the Qiagen miRNeasy Mini kit (Valencia, CA) according to manufacturer’s instructions. Extracted total RNA was treated with RNase-Free DNase I (Qiagen, Valencia, CA), quantitated by UV spectrophotometry, and quality checked by a 1% denaturing agarose gel as previously described [10]. Five micrograms of total RNA was converted to cDNA in an 100 µl PCR reaction using random primers and Multiscribe reverse transcriptase (Applied Biosystems, Foster City, CA). mRNA expression was assessed using TaqMan Gene Expression Assays and master mix (Applied Biosystems, Foster City, CA) detected by an ABI 7300 Real-Time PCR system as described previously [10]. Gene expression values were quantified by comparing C_T_ values of the unknown sample to the gene-specific standard curve and normalized to the expression of beta-actin.

**Figure 6:**
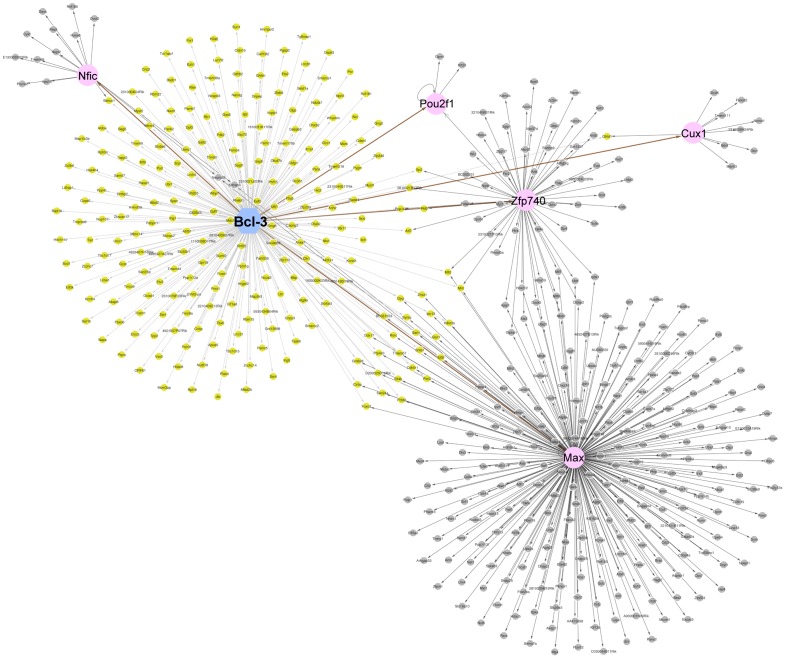
Network of direct and indirect Bcl-3 target genes during unloading. Display of ChIP-Array data from a comparison of 2,817 Bcl-3 binding peaks increased in unloading over controls vs. 3,334 genes with increased expression in unloaded muscles. A blue circle indicates the location of Bcl-3. Projections from Bcl-3 in yellow are the direct targets including 5 direct transcription factor target genes, which are indicated in pink circles. Projections from pink targets are indirect Bcl-3 targets indicated in gray. Thus, ChIP-Array found 241 direct targets, 5 direct targets with indirect targets (the transcription factors) and 305 indirect target genes of Bcl-3 in the gene expression array data. The direct targets are those from the Bcl-3 ChIP-seq list with some not identified because of gene name terminology differences in the ChIPArray format. However the direct targets with indirect targets yields important evidence for a hierarchy of gene regulation.

### Microarray Processing and Analysis

Whole-genome gene expression profiling experiments were carried out by the Boston University Microarray Core Facility. Each group (control and unloaded) included 4 independent total RNA samples with a minimal RIN number 8.0 verified by Bioanalyzer 2100 (Agilent Technology, Palo Alto, CA). Each total RNA sample was amplified, labeled, and hybridized on a mouse Affymetrix Gene 1.0 ST array (Santa Clara, CA) per manufacture instructions to measure expression of 28,853 well-annotated genes. A total of 8 array images were acquired by GeneChip Scanner 3000 7G and quality assessed by Affymetrix Expression Console (Santa Clara, CA). Gene expression signals were generated by robust multi-array analysis (RMA) [16] using Brainarray MoGene 1.0ST custom CDF files [17]. Differential gene expression was computed using the Comparative Marker Selection module in Genepattern database (Broad Institute, Cambridge, MA) which compares mean differences between control and unloaded groups by two-way parametric t-test. *P*-value ≤0.05 and *q*-value ≤0.05 were used to identify genes that were significantly differentially expressed with hind limb unloading. The microarray data reported in this paper have been deposited in the NCBI Gene Expression Omnibus (GEO) with accession no. GSE40578.

**Figure 7:**
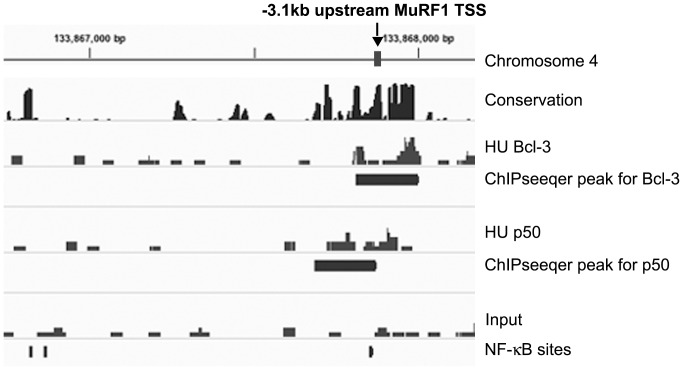
Bcl-3 and p50 binding profile at MuRF1 locus. An assembly of ChIP-seq data visualized by IGV for the Trim63/MuRF1 gene. The top line is a representation of the genomic size and location of the region of chromosome 4. Vertical ticks are 500 bp apart. The region of this gene (MuRF1) labeled is 3.1 kb 5′ of the TSS. The next rows are labeled as follows: Conservation, the track of Phastcons for sequence similarity among placental mammals; HU Bcl-3, a representation of the.sam alignments for the Bcl-3 ChIPseq of unloaded muscle; ChIPseeqer peak for Bcl-3, black horizontal bar indicates the location of the statistically-qualified peak of sequencing alignments called by the ChIPseeqer algorithm; HU p50, a representation of the.sam alignments for the p50 ChIPseq of the hindlimb unloaded muscle; ChIPseeqer peak for p50, black horizontal bar indicates the location of the statistically-qualified peak of sequencing alignments called by the ChIPseeqer algorithm; Input, a representation of the.sam alignments for the non-ChIP unloaded chromatin; NF-κB sites, location for the 3 JASPAR identified NF-κB consensus sites in this region and in our reporter construct.

### Plasmids and Site Directed Mutagenesis

The mouse MuRF1 promoter luciferase plasmid which contains 4.4 kb of the 5′ upstream MuRF1 promoter region was a gift from S. Shoelson [18]. *In silico* analysis of transcription factor binding sites in this 4.4 kb MuRF1 promoter region was performed by Clover [19] which identified 3 putative NF-κB sites in the 5′ 2 kb of the cloned promoter fragment. The 2 kb MuRF1-luc deletion construct was created by cutting the MuRF1-luc plasmid with NheI and SmaI, and ligating blunted ends to remove the 5′ 2 kb of MuRF1 promoter sequence. This produced a promoter without the 3 putative NF-κB sites. Also using the 4.4 kb MuRF1 promoter, site directed mutagenesis was used to mutate all 3 putative NF-κB sites of MuRF1-luc using PCR primers designed by the QuikChange Primer Design Program (Agilent, Santa Clara, CA). The oligonucleotides were designed in our lab and then made by Invitrogen (Carlsbad, CA). The target sequences are listed with the NF-κB site underlined and the mutated nucleotides capitalized: κB1 5′-caa act ctc agg ttt ctg aaa agt GAG ttt tct agt gac aat ccc aaa gag-3′, κB2 5′- ccc aaa gag cac aga ctt aCT Caa gtt cca gcg cta cca g-3′, κB3 5′- ccg ccc atg tgg gaa ctt GAG cat ctc acc ctt tga ctt-3′. A reaction was performed by mixing 100 ng of each phosphorylated primer, 100 ng MuRF1-luc, 1.25 U *Pfu*Ultra High-fidelity DNA polymerase (Agilent), and 20 U *Taq* DNA ligase (New England Biolabs, Ipswich, MA) and then the PCR was carried out in a thermal cycler set as follows: 95°C for 2 min (denature), 30 cycles of 95°C for 50 sec, 60°C for 50 sec, and 68°C for 5 min, and followed by a final incubation at 68°C for 5 min (extension). After DpnI treatment, amplified PCR products were transformed into XL10-Gold Ultracompetent bacteria (Agilent) according to manufacturer’s instructions. The DNA sequences of the wild type MuRF1 reporter, MuRF1 deletant, and the MuRF1 3 κB mutant constructs were verified by Genewiz sequencing services (South Plainfield, NJ).

**Figure 8:**
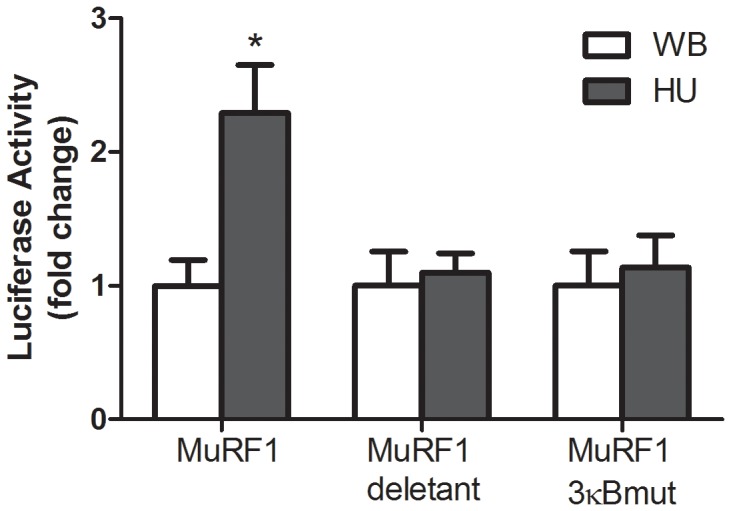
Luciferase activity of MuRF1 reporter constructs in weight bearing vs. unloaded muscle. Reporter constructs were electroporated into rat soleus and then unloaded for 5 days. MuRF1 is 4.4 kb promoter region of mouse MuRF1 driving expression of luciferase. MuRF1 deletant is a plasmid containing the 4.4 kb of the MuRF1 promoter but the distal 2 kb of the promoter was excised (from the 4.4 kb promoter) thus removing all 3 κB sites, and MuRF1 3κBmut is a plasmid containing the 4.4 kb of the MuRF1 promoter but the 3 NF-kB binding sites were mutated. * indicates statistical difference compared to weight bearing (WB) (*P*<0.05).

### Luciferase Assay

Soleus muscles transfected with plasmid DNA were homogenized in 1 mL passive lysis buffer (Promega, Madison, WI). Homogenates were centrifuged at 5,500 g at 4°C for 20 min. Supernatant was collected, diluted 1∶20, and mixed with 100 µl luciferase assay reagents (Promega). Luciferase activity was measured by a TD-20/20 illuminometer (Turner Designs Inc), which reflected total muscle luciferase activity.

### Statistical Analysis

For RT-qPCR and luciferase activity, a two-tailed independent t-test was performed to determine statistical significance between WB and HU groups. A *P* value less than 0.05 was considered statistically significant.

## Results

### Characterization of Bcl-3 ChIP-seq

Since transcriptional activation of the p50-Bcl-3 complex will not happen without Bcl-3 [11] we reasoned that its binding is the best for following the active NF-kB complex in unloaded muscle at a genome-wide level. Bcl-3 ChIP sequences from unloaded muscle were put through the peak finding algorithm of ChIPseeqer [15], which identifies peaks with increased Bcl-3 binding compared to weight bearing muscle. By using a low stringency peak height cutoff, 49,000 Bcl-3 peaks were found. These peaks were evenly distributed across the mouse genome (Figure S1). Using a web based tool called Nebula [20], [21] a component of the Galaxy suite of programs, the distribution of Bcl-3 peaks from unloaded muscle were compared to random peaks found from the input fraction of chromatin (Figure 1). This showed that the Bcl-3 ChIP had succeeded in enriching many Bcl-3 binding sites (i.e., peaks) near the activation sites of transcription across the entire genome.

We then took the sequence alignments from unloaded muscle Bcl-3 ChIP-seq and compared them to weight bearing muscle sequences in order to find peaks that were at least 2-fold increased using the peak finder in ChIPseeqer. By using this level of stringency for peak finding we obtained 2,817 Bcl-3 peaks in unloaded compared to weight bearing muscle. Phastcon analysis using the Cistrome/Galaxy program [21] was used to show that the peaks were located at phylogenetically conserved sites (Figure 2). Annotation of the parts of genes associated with unloading-induced peaks showed that they were mainly in promoters (Figure 3). We then focused on the peaks in the promoters of the genes found, from −4 to +2 kb relative to the TSS (n = 845).

### Gene Ontology Terms Identified by Genome-wide Increased Bcl-3 Binding to Promoter Regions in Unloaded Muscle

To find the important functional groups of genes that show increased Bcl-3 binding with muscle unloading, we evaluated the peaks found in unloaded compared to control muscle for gene ontology terms/pathways. To do this we used the iPAGE algorithm, a module of the ChIPseeqer set of computational programs (Figure 4). iPAGE was set up to restrict its analysis to the 845 peaks (out of the 2,817 total) which were found in promoters (−4 kb to +2 kb relative to TSS). As with any gene ontology (GO) mapping algorithm, iPAGE identifies GO terms in which the peaks found are statistically over-represented relative to calculations for random distribution. The 23 GO terms that were found for genes containing Bcl-3 peaks in unloaded muscle were from three biological processes: protein catabolism, development/differentiation and sugar/glucose metabolism. There were 24 genes found in the 23 GO pathways and these are presented in Table 1.

The most abundant group with 14 genes in 11 GO pathways was for protein catabolism. The genes are ones that function in several aspects of catabolism in muscle including several E3 ligases of the ubiquitin proteasome pathway, and importantly, two genes that contribute to the cell catabolism driven by the N-end rule. Those genes are Ubr1/E3α, the N-end recognin E3 ligase, and Ate1, the arginyltransferase responsible for modifying several amino acid amino termini for Ubr1 recognition. The sequence alignments and locations for the peaks for these two genes have been visualized by use of Integrative Genomics Viewer (IGV) [22], (http://www.broadinstitute.org/igv/) and are shown in Figure 5. For both genes, a Bcl-3 peak due to unloading was identified at an evolutionary conserved region close to the TSS and was in close proximity to a JASPAR matrices defined NF-κB site. In addition, data for ChIP-seq with p50 antibodies showed p50 binding at or very near the peak sites of Bcl-3 binding (data not shown). Another E3 ligase found was Trim63/MuRF1, a muscle specific protein thought to target heavy myosin chains during atrophy [23], [24].

Also found in the GO pathways and shown in Table 1 are genes that function in the reduction of reactive oxygen species, including SOD1, and several phosphatases. The other GO terms having genes represented are those involved in regulating myogenesis, particularly in the Wnt pathway, and those in glucose metabolism, including glycogen phosphorylase and 7-phosphofructokinase, genes that liberate glucose and control its glycolytic metabolism respectively. Several of the genes, especially the E3 ligases found as Bcl-3 targets by ChIP-seq were subject to qPCR to verify gene activation during unloading and these data are shown in Table 2.

The advantage of iPAGE is that it can find the most important functions of the overrepresented genes having peaks with unloading in an unbiased fashion. However, there are other genes with Bcl-3 peaks in the promoter region that are likely to be important to atrophy. For example, several proteolytic pathway genes not identified by iPAGE also show Bcl-3 peaks with unloading (Psmc1, Psmb7, Ube2b, Ubb, Cul4a, Rnf135, Rnf13, Atg3). For transcription factors, Foxo1, Foxo3, and Cebpa show peaks as well as several translation initiating genes including Eif4b and Eif3f. All of the genes with unloading-induced increased Bcl-3 binding in their promoters are listed in Table S1.

### Direct and Indirect Targets of Bcl-3

Since we were interested in further describing direct and indirect targets of the Bcl-3 transactivator at the genome-wide level, we used the algorithms of ChIPArray [25] to bring together our ChIPseq data on Bcl-3 binding to promoters with the genes whose mRNA was upregulated as determined by global gene expression array (28,853 transcripts) of control vs. unloaded muscle (Figure 6). ChIPArray found 241 direct targets, 5 direct targets with indirect targets (transcription factors) and 305 indirect target genes of Bcl-3. The indirect target genes, according to this analysis, are controlled by the direct Bcl-3 targeted transcription factors Max, Zfp740, Nfic, Cux1 and Pou2f1. Max appears to regulate the largest number of indirect target genes.

### Testing a Bcl-3 Binding Region in Gene Activation

In a previous paper, we found genes to be direct or indirect targets of Bcl-3 based on gene expression in unloaded muscle from wild type vs. Bcl3 knockout mice. We selected several of these genes for further study that were thought to be involved with the atrophy process. We identified NF-kB sites in these genes in silico and we found ChIP-PCR support for increased Bcl-3 binding [10]. One of these genes, MuRF1, had three in silico NF-kB sites in the 4.4 kb region of the promoter that had already been cloned into a luciferase reporter [18]. The present study identified MuRF1 by iPAGE as being a Bcl-3 target in the GO categories of proteolysis. The data identified a peak at one of the in silico-identified NF-kB sites of the MuRF1 promoter. The alignments for the Bcl-3 binding site in the MuRF1 promoter are shown in Figure 7. Data for ChIP-seq with p50 antibodies are also presented, indicating the associated binding of p50 and a location for a ChIPseeqer peak very close to the peak of Bcl-3 binding.

In weight bearing and unloaded muscle, we compared the MuRF1 promoter-reporter activity from the 4.4 kb promoter and a smaller MuRF1 reporter in which the 5′ end was deleted by removing the 2 kb upstream region containing all 3 NF-kB sites. We also compared a MuRF1 reporter in which site mutagenesis was used to abolish the 3 NF-kB sites located at −3.1, −4.1, and −4.2 kb of the 4.4 kb promoter (Figure 8). From these plasmids we found that removal of the entire region containing NF-κB sites completely abolished the increase in reporter activity due to unloading, and specifically, that the decrease in activity is dependent on NF-kB sites. A further test of the NF-kB-dependent effect of Bcl-3 on the activity of the MuRF1 promoter was carried out in vitro. Although not as complete as the effect in vivo, it is clear in cell culture that mutation of the NF-kB sites alone is sufficient to reduce Bcl-3 induction of the MuRF1 gene (Figure S2).

## Discussion

The location of unloading-induced Bcl-3 binding in promoters across the genome demonstrates a remarkable molecular genetic association between this NF-kB transcription factor and the atrophy process in unloaded muscle. The most impressive finding is the degree to which protein catabolic pathways were targeted by this Bcl-3 regulatory network. The impartial gene ontology algorithm called iPage was able to indicate that the major part of the over-represented genes with Bcl-3 peaks due to unloading were involved in protein degradation or its signaling. Of the 23 GO terms found, 11 were catabolic. In those groups there were 14 genes. Six of these genes were E3 ubiquitin protein ligases. Of interest is that one of the E3s is Ubr1, the gene also known as E3α ligase. It is one of the major recognition ligases for ubiquitinating proteins that have destabilizing amino acids at their N termini. It is noted in the literature that the ubiquitination present in atrophy is largely due to the activation of the N-end rule pathway [26], [27]. A knockout of Ubr1 shows muscle specific abrogation of N-end rule ubiquitination [28]. Another target gene of Bcl-3 and the N-end rule pathway is arginyltransferase, the enzyme encoded by the Ate1 gene, which puts an arginine destabilizing amino acid on the amino termini populated by aspartic and glutamic acids and by oxidized cysteine [29]. In addition to the ChIP-seq data, the mRNA for these N-end rule genes was found to be upregulated in unloading. The other catabolic proteins fall in all major families of protein degradation including lysosomal (Ppt1) and oxidative pathways (Sod1).

For one E3 ubiquitin ligase, MuRF1, we investigated the importance of the NF-kB sites in the promoter region, found by our ChIP-seq data and by in silico analysis, with MuRF1 promoter-reporter activity due to muscle unloading. Deletion of the distal 2 kb region of the 4.4 kb MuRF1 promoter construct contained all the putative NF-kB sites. Unloading induced activation of MuRF1 was abolished in this deletant MuRF1 reporter. The remaining 2.4 kb of the proximal MuRF1 promoter contains consensus sites for other factors such as Foxo (not shown) suggesting that it is not required for unloading regulation of MuRF1. We found that consistent with our ChIP-seq binding data, the mutagenesis of NF-kB sites also eliminated unloading-induced activation of the MuRF1 reporter.

A number of GO pathways identified in our results are involved with glucose metabolism, and the genes include phosphofructokinase, the rate limiting enzyme of the glycolysis pathway, and muscle glycogen phosphorylase, the enzyme responsible for liberating glucose from muscle glycogen stores. In a separate study, phosphofructokinase was found upregulated in unloaded rat muscles, reflecting a change to increased glycolysis and use of glycogen stores with disuse [30]. Two other glycolytic genes, not part of the iPage results, also showed Bcl-3 peaks in their promoters due to unloading, hexokinase (HK2) and aldolase A (AldoA). Finally, the GO terms include 7 involved in development and morphogenesis. The genes from these pathways include two affecting the Wnt pathway (Tcf7l2 and Apc). The interest in Tcf7l2 (also known as Tcf4) is recently heightened as it is thought to be significantly linked to type II diabetes, which is characterized, by insulin resistance and changes in glucose metabolism, especially in muscle [31]. Apc, acts as a Wnt antagonist with direct effects on Tcf7l2 [32]. Psap is a precursor of the saposins which regulate lysosomal degradation of sphingolipids. Sphingolipids appear to be directly involved in both muscle atrophy [33] and insulin resistance [34].

In order to further explore the combination of our ChIP-seq data and that from our extensive work on the changes in gene expression with unloading, we used the network-available algorithms for ChIP and expression array analysis available from ChIPArray [25] (http://wanglab.hku.hk/ChIP-Array). Previously we postulated that our results from gene expression arrays for unloading in wild type vs. Bcl3 knockout mice had indicated a set of indirect and direct targets. We felt that the use of ChIP-seq would determine, by showing binding of Bcl-3 to complexes on the target genes, that these were direct targets. With that accomplished we knew that some of the direct targets of Bcl-3 should be the factors that cause the gene expression array changes in the indirect targets. This is difficult to determine by searching within the results of ChIP-seq, but ChIPArray is able to show these relationships. From the ChIP-Array results we have found 5 new candidate transcription factors, most notably including Max, that appear to extend the Bcl-3 gene activation network in muscle atrophy.

We have provided, in the plots of sequence alignments and peaks, the location of alignments for p50. It is thought that Bcl-3 binds to DNA by an association with p50 or p52 homodimers [35]. We have not determined the requirement for p52 in unloading and although it is expressed in muscle, its localization to the nucleus does not change with disuse [7]. On the other hand, p50 is required for disuse atrophy [8], and, we found that an estimation of the p50 gene targets in muscle unloading are a subset of those for Bcl-3 [10]. Therefore, it is likely that there are some associations of Bcl-3 and targets that are not due to p50 and perhaps these are due to binding to p52 homodimers instead. A simple evaluation of p50 peaks with unloading and weight bearing did not produce the same iPage results as for Bcl-3 (not shown). However this is not surprising for two reasons. First, the activity of p50:Bcl-3 complexes resides in Bcl-3. Therefore it would not matter to Bcl-3 activity whether p50 was increased or simply present in both the weight bearing and unloaded conditions. Second, the dimer between p50 and p65 would also be picked up by our ChIP-seq when using the p50 antibody for ChIP. Therefore when attempting to study gene ontology mapping for p50 we would be looking at complexes with Bcl-3 and those with p65. We know that p65 in the nucleus does not change with unloading [7] and kB sites in promoters of 14 upregulated atrophy genes do not showed increased p65 binding by ChIP-PCR [10], but it is likely that there are p50:p65 heterodimers in the nucleus maintaining homeostatic gene activities. Therefore the GO terms associated with p50 peaks would be a list of pathways, many not having to do with the atrophy under study.

### Conclusion

With this first assay of the binding of Bcl-3 to promoter regions of genes during muscle atrophy it is quite clear that Bcl-3 regulates transcriptional networks of the genes involved in muscle catabolism and metabolism. These data provide the molecular evidence to explain why Bcl-3 knockout mice do not show unloading atrophy. Our data describe Bcl-3 as a global regulator both of the proteolysis and the change in energy metabolism that are essential components of muscle atrophy due to disuse. We have identified for the first time, gene networks that are determined by the binding of a transcription factor (Bcl-3) that is required for muscle atrophy. Gene networks of other transcription factors involved in disuse and other causes of muscle atrophy are yet to be identified but their study, using methods similar to the ones we have pioneered here, will be of great interest in order to complete our understanding of the molecular biology of skeletal muscle atrophy.

## Supporting Information

Figure S1 **Galaxy/Cistrome-found distribution of Bcl-3 peaks over the mouse genome for each chromosome.** Red vertical lines show the peak heights and indicate the low stringency 49,000 Bcl-3 peaks that were greater in unloaded vs. control muscle. The position of each peak is plotted from the beginning to end of the chromosome with scale indicated in base pairs by a ruler at the bottom of the graph.
(TIF)

Figure S2 **A graph of the results of transfecting MuRF1-luciferase reporter plasmids into Bcl3−/− fibroblasts with and without addition of a Bcl-3 expression vector.** A cell line of fibroblasts was isolated from the gastrocnemius muscles of a Bcl3 knockout mouse by enzyme dissociation. The cells were transfected with Effectene (Qiagen) and luciferase activity was measured after 48 hours. Luciferase activity is induced by 11 fold when Bcl-3 is supplemented to the reporter-transfected cells, while mutagenesis of the three NF-κB sites in that reporter reduces this induction by 40%.
(TIF)

Table S1 **A table of the 858 genes with ChIPseeqer-defined Bcl-3 binding peaks in their promoter regions (−4** **kb to +2** **kb relative to TSS).** The 845 peaks in promoters from the 2817 total peaks of unloading vs. control Bcl-3 binding map to 858 genes since some peaks are within guideline distances of the TSS of two genes. A cursory assignment of gene categories and functions was carried out using the Gene database of NCBI (http://www.ncbi.nlm.nih.gov/sites/entrez?db=gene). The columns are A, Functional Category (in the broad sense); B, Gene Symbol; C, Gene Function Description; and D–H supplemental and alternative functional information for some of the genes.
(XLSX)
