# Supplementary figures and images for: The ChIP-seq-Defined Networks of Bcl-3 Gene Binding Support Its Required Role in Skeletal Muscle Atrophy
Source: PLoS One. 2012 Dec 10;7(12):e51478. doi: 10.1371/journal.pone.0051478 (PMC3519692; doi:10.1371/journal.pone.0051478)

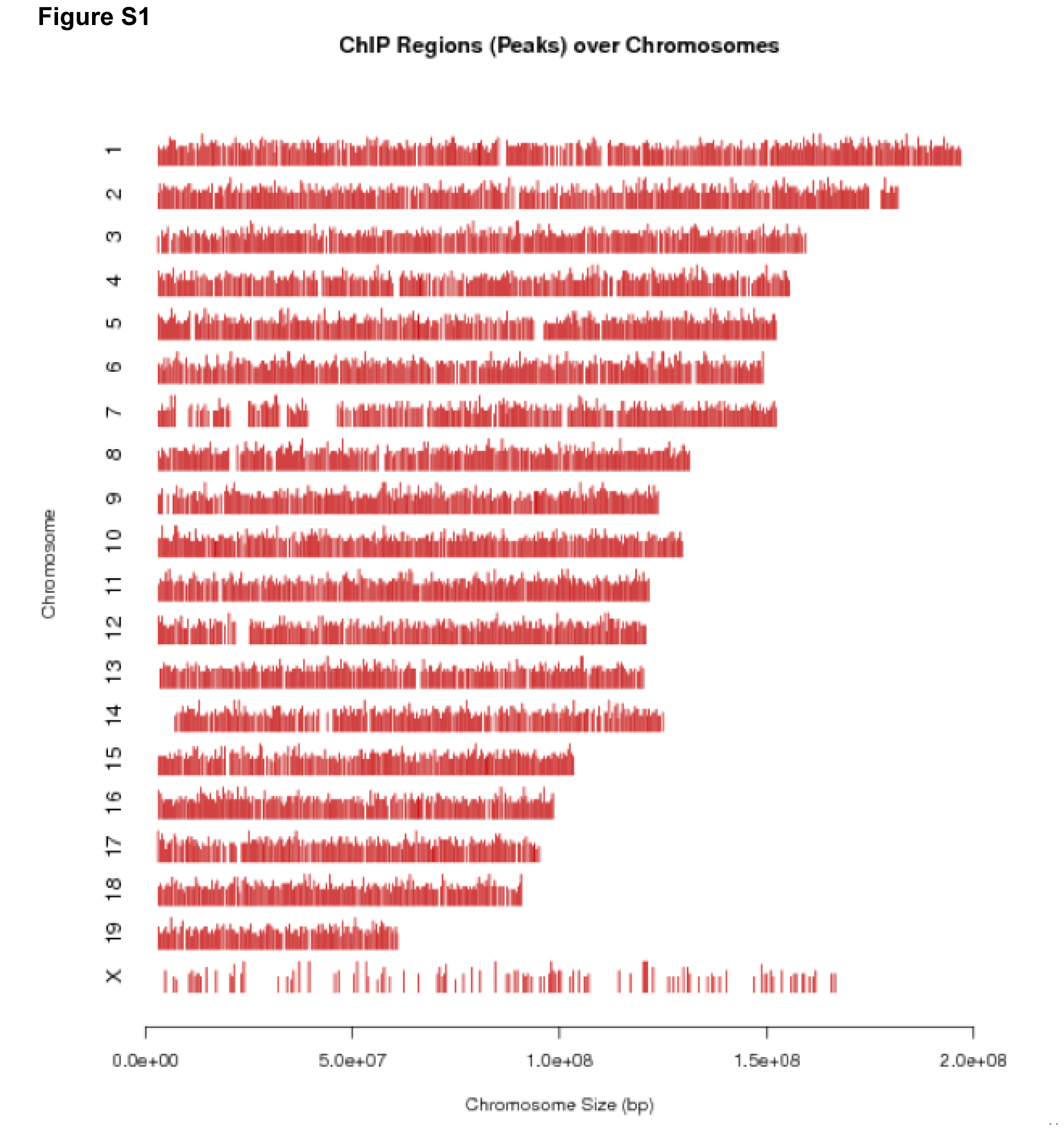

Supplement: Figure S1 — Galaxy/Cistrome-found distribution of Bcl-3 peaks over the mouse genome for each chromosome. Red vertical lines show the peak heights and indicate the low stringency 49,000 Bcl-3 peaks that were greater in unloaded vs. control muscle. The position of each peak is plotted from the beginning to end of the chromosome with scale indicated in base pairs by a ruler at the bottom of the graph. (TIF) [file pone.0051478.s001.tif]

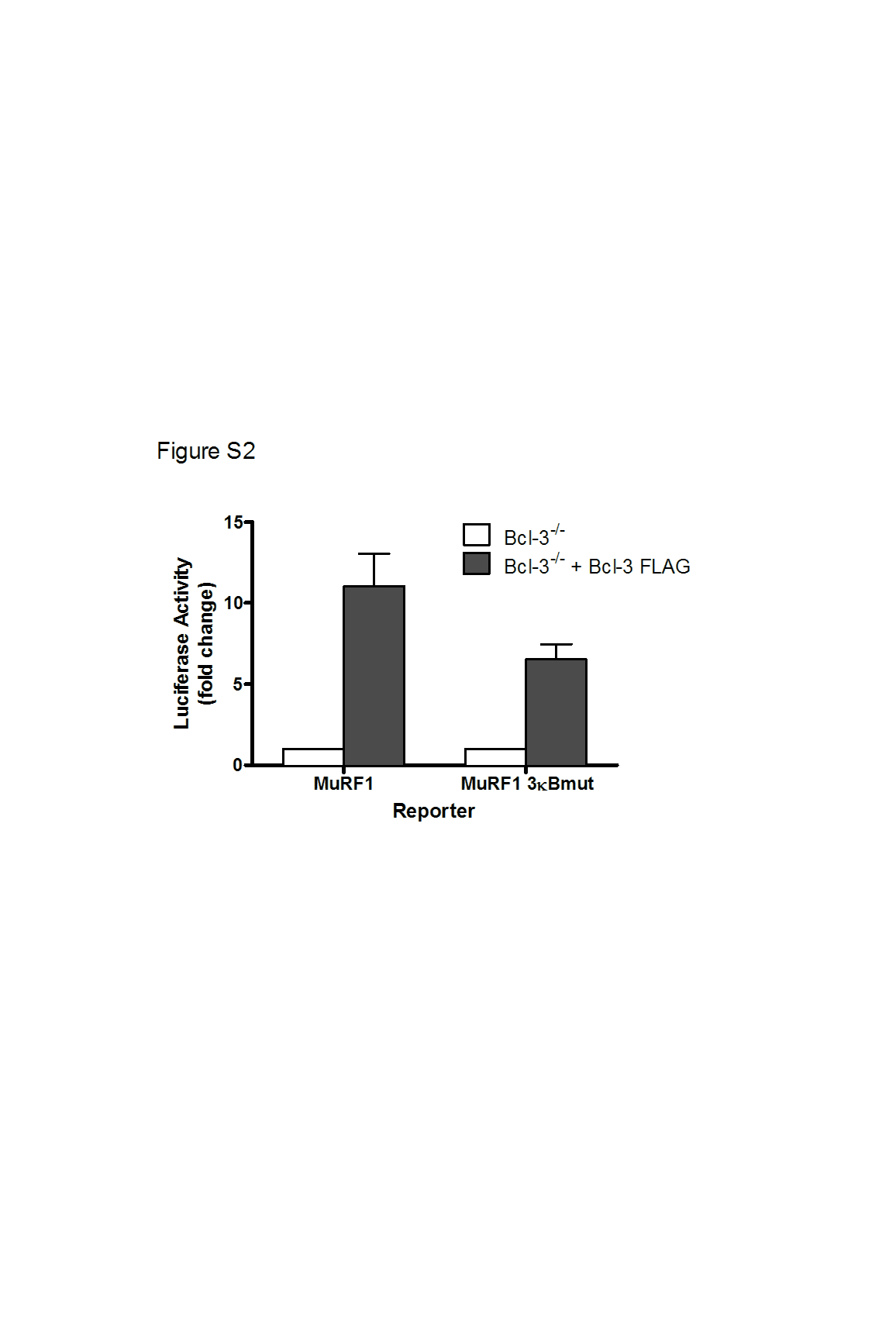

Supplement: Figure S2 — A graph of the results of transfecting MuRF1-luciferase reporter plasmids into Bcl3−/− fibroblasts with and without addition of a Bcl-3 expression vector. A cell line of fibroblasts was isolated from the gastrocnemius muscles of a Bcl3 knockout mouse by enzyme dissociation. The cells were transfected with Effectene (Qiagen) and luciferase activity was measured after 48 hours. Luciferase activity is induced by 11 fold when Bcl-3 is supplemented to the reporter-transfected cells, while mutagenesis of the three NF-κB sites in that reporter reduces this induction by 40%. (TIF) [file pone.0051478.s002.tif]
